# Fisetin Induces Apoptosis Through p53-Mediated Up-Regulation of DR5 Expression in Human Renal Carcinoma Caki Cells

**DOI:** 10.3390/molecules22081285

**Published:** 2017-08-02

**Authors:** Kyoung-jin Min, Ju-Ock Nam, Taeg Kyu Kwon

**Affiliations:** 1 Department of Immunology, School of Medicine, Keimyung University, 2800 Dalgubeoldaero, Dalseo-Gu, Daegu 704-701, Korea; kyoungjin.min@gmail.com; 2 Department of Food Science and Biotechnology, Kyungpook National University, Daegu 41566, Korea; namjo73@gmail.com

**Keywords:** fisetin, apoptosis, p53, DR5, renal carcinoma

## Abstract

Fisetin is a natural compound found in fruits and vegetables such as strawberries, apples, cucumbers, and onions. Since fisetin can elicit anti-cancer effects, including anti-proliferation and anti-migration, we investigated whether fisetin induced apoptosis in human renal carcinoma (Caki) cells. Fisetin markedly induced sub-G1 population and cleavage of poly (ADP-ribose) polymerase (PARP), which is a marker of apoptosis, and increased caspase activation. We found that pan-caspase inhibitor (z-VAD-fmk) inhibited fisetin-induced apoptosis. In addition, fisetin induced death receptor 5 (DR5) expression at the transcriptional level, and down-regulation of DR5 by siRNA blocked fisetin-induced apoptosis. Furthermore, fisetin induced p53 protein expression through up-regulation of protein stability, whereas down-regulation of p53 by siRNA markedly inhibited fisetin-induced DR5 expression. In contrast, fisetin induced up-regulation of CHOP expression and reactive oxygen species production, which had no effect on fisetin-induced apoptosis. Taken together, our study demonstrates that fisetin induced apoptosis through p53 mediated up-regulation of DR5 expression at the transcriptional level.

## 1. Introduction

Many natural flavonoids have anti-cancer effects. Fisetin (3′,4′,7-tetrahydroxyflavone) is one of these natural compounds, found in fruits and vegetables such as strawberries, apples, cucumbers, and onions [1]. Fisetin has multiple biological effects, such as anti-proliferation [2,3] and anti-inflammation activities [4,5]. Additionally, fisetin has anti-cancer effects in several types of cancer cells [3,6,7]. For example, fisetin induces apoptosis via inhibition of the MAPK signaling pathway in human non-small cell lung cancer [7] and reactive oxygen species (ROS) production in human oral cancer cells [8]. Furthermore, fisetin has a sensitizing effect in anti-cancer therapy. Combined treatment with fisetin and cabazitaxol has been shown to induce apoptosis [9]. Additionally, treatment with fisetin enhanced tumor necrosis factor-related apoptosis-inducing ligand (TRAIL)-mediated apoptosis in cancer cells [10]. However, the molecular mechanism of this anti-cancer effect by fisetin is not well understood.

Apoptosis signaling is mainly divided into two pathways: the intrinsic and the extrinsic pathway. The intrinsic pathway is activated from within the cell, with the Bcl-2 family of proteins playing a critical role on apoptosis. In contrast, the extrinsic pathway is activated from outside of the cell with the binding of a ligand to death receptors (DRs) on the membrane, creating a critical event. These TRAILs bind to their receptor (DR4 or DR5), and recruit caspase-8 into a death-inducing signaling complex (DISC). Activation of caspase-8 induces apoptosis. DR4 or DR5-mediated apoptosis is modulated by two main steps: up-regulation of DR expression and expression of DRs on the cell membrane. First, up-regulation of DR expression at the transcription level plays a critical role on apoptosis. The promoter for the DR4 and DR5 genes contains binding elements for transcription factors, including activator protein (AP) 1 [11], p53 [12,13], C/EBP homologous protein (CHOP) [14], and nuclear factor-κB (NF-κB) [15]. Second, the surface expression of DRs on the cell plasma membrane also modulates apoptosis. In some cancer cells, DRs remain within autophagosomes, nuclei, nuclear membranes, or the endoplasmic reticulum (ER), resulting in down-regulation of DR expression on the cell surface, leading to drug resistance [16,17,18,19,20]. The presentation of DRs on the cell surface overcomes drug resistance.

Renal cell carcinoma is the most common form of kidney cancer, and is one of the drug-resistant malignancies in humans, which is a frequent cause of cancer mortality [21]. Therefore, there is a necessity for the development of novel therapeutic strategies against this type of cancer. In our study, we examined the effects of fisetin on apoptosis, and investigated the molecular mechanism in fisetin-induced apoptosis in renal cancer cells.

## 2. Results

### 2.1. Fisetin Induces Apoptosis in Human Renal Carcinoma Caki Cells

**Figure 1:**
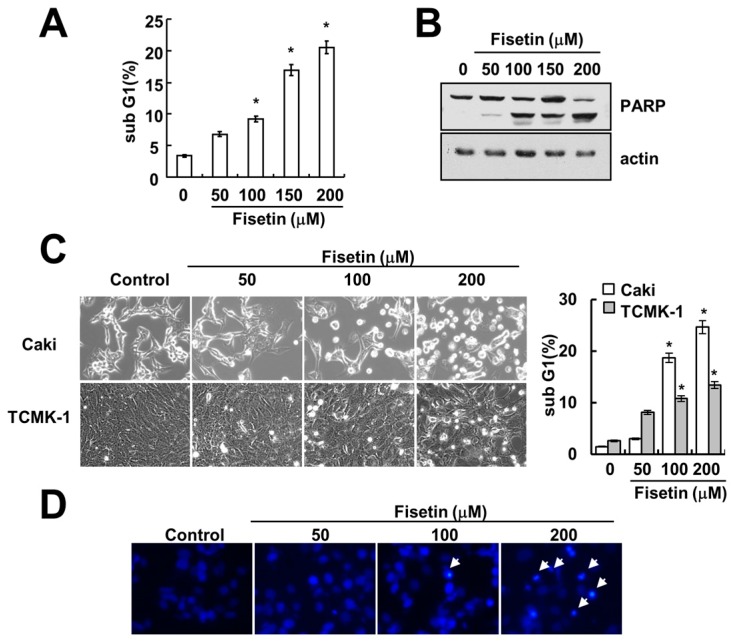

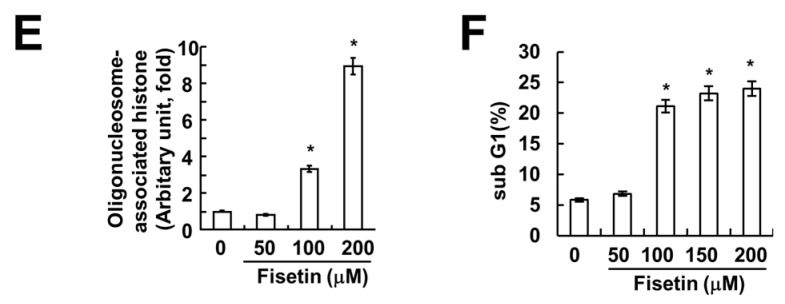
Fisetin induces apoptosis in human renal carcinoma Caki cells. (**A**,**B**) Caki cells were treated with the indicated concentrations of fisetin for 24 h; (**A**) The sub-G1 fraction was measured using flow cytometry as an indicator of the level of apoptosis; (**B**) The protein expression levels of PARP and actin were determined by western blotting; (**C**) Caki cells and TCMK-1 cells were treated with the indicated concentrations of fisetin for 24 h. The cell morphology was examined using interference light microscopy. The sub-G1 fraction was measured by flow cytometry; (**D**,**E**) Caki cells were treated with the indicated concentrations of fisetin for 24 h; (**D**) The condensation and fragmentation of the nuclei were detected by 4′,6′-diamidino-2-phenylindole staining; (**E**) The cytoplasmic histone-associated DNA fragments were determined by a DNA fragmentation detection kit; (**F**) SK-Hep-1 cells were treated with the indicated concentrations of fisetin for 24 h. The sub-G1 fraction was measured using flow cytometry. The values in (**A**,**C**,**E**,**F**) represent the mean ± SD from three independent samples. * *p* < 0.01 compared to the control.

Previous studies have reported that fisetin has anti-cancer effects, such as induction of apoptosis [7] and inhibition of proliferation [3]. Therefore, we investigated whether fisetin induces apoptosis in human renal carcinoma (Caki) cells. As shown in Figure 1A, treatment with fisetin induced a sub-G1 population in a dose-dependent manner. Furthermore, cleavage of poly(ADP-ribose) polymerase (PARP)—a substrate of caspase and a marker of apoptosis—was also increased (Figure 1B). Fisetin induced morphological changes, followed by cell shrinkage and membrane blebbing (Figure 1C). We also investigated the effect of fisetin on apoptosis in normal mouse kidney (TCMK-1) cells. Fisetin induced apoptosis in TCMK-1 cells, however the levels of apoptosis were lower, compared with those of Caki cells (Figure 1C). Therefore, this data suggested that cancer cells were more sensitive than normal cells to fisetin treatment. In addition, chromosomal damage and DNA fragmentation was also detected in fisetin-treated cells (Figure 1D,E). We next investigated the effect of fisetin on apoptosis in a second type of cancer cell: human hepatoma SK-Hep1 cells. Fisetin markedly induced apoptosis in SK-Hep1 cells (Figure 1F). Therefore, this data indicated that fisetin induced apoptosis in cancer cells.

### 2.2. Caspase Activation Is Involved in Fisetin-Induced Apoptosis

**Figure 2:**
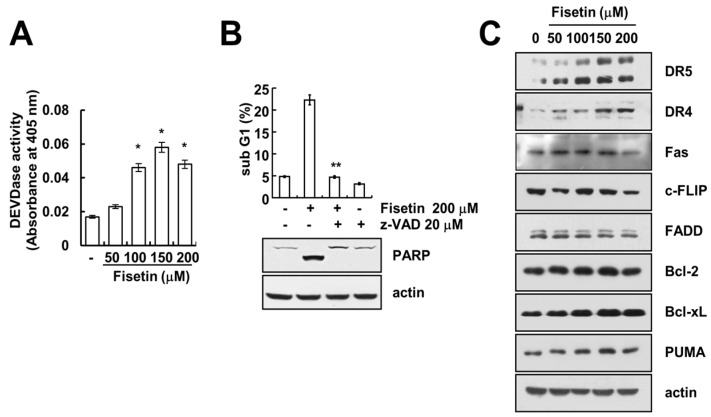
Fisetin induced apoptosis in a caspase-dependent manner. (**A**) Caki cells were treated with the indicated concentrations of fisetin for 24 h. Caspase activities were determined with colorimetric assays using caspase-3 (DEVDase) assay kits; (**B**) Caki cells were treated with 200 μM fisetin in the presence or absence of 20 μM z-VAD-fmk (z-VAD). The sub-G1 fraction was measured by flow cytometry. The protein expression levels of PARP and actin were determined by Western blotting. The level of actin was used as a loading control; (**C**) Caki cells were treated with the indicated concentrations of fisetin for 24 h. The protein expression levels of DR5, DR4, Fas, c-FLIP, FADD, Bcl-2, Bcl-xL, PUMA and actin were determined by western blotting. The level of actin was used as a loading control; the values in (**A**,**B**) represent the mean ± SD from three independent samples. * *p* < 0.01 compared with the control. ** *p* < 0.01 compared with the fisetin treatment.

Caspase activation plays a critical role in apoptosis. As such, we investigated whether fisetin-induced apoptosis is dependent upon the activation of caspase. Fisetin markedly increased caspase activation (Figure 2A). Moreover, z-VAD-fmk, a pan-caspase inhibitor, completely blocked fisetin-induced sub-G1 population and PARP cleavage (Figure 2B). This data suggested that fisetin induced caspase-mediated apoptosis. Next, to identify the molecular mechanism of fisetin-induced apoptosis, we examined the expression of apoptosis-related proteins. 

As shown in Figure 2C, the expression levels of Fas, c-FLIP, FADD, Bcl-2, Bcl-xL, and PUMA did not change with fisetin treatment (Figure 2C). However, fisetin induced up-regulation of death receptor DR4 and DR5 expression in a dose-dependent manner (Figure 2C).

### 2.3. Fisetin Induced Apoptosis Through Up-Regulation of DR5 Expression

**Figure 3:**
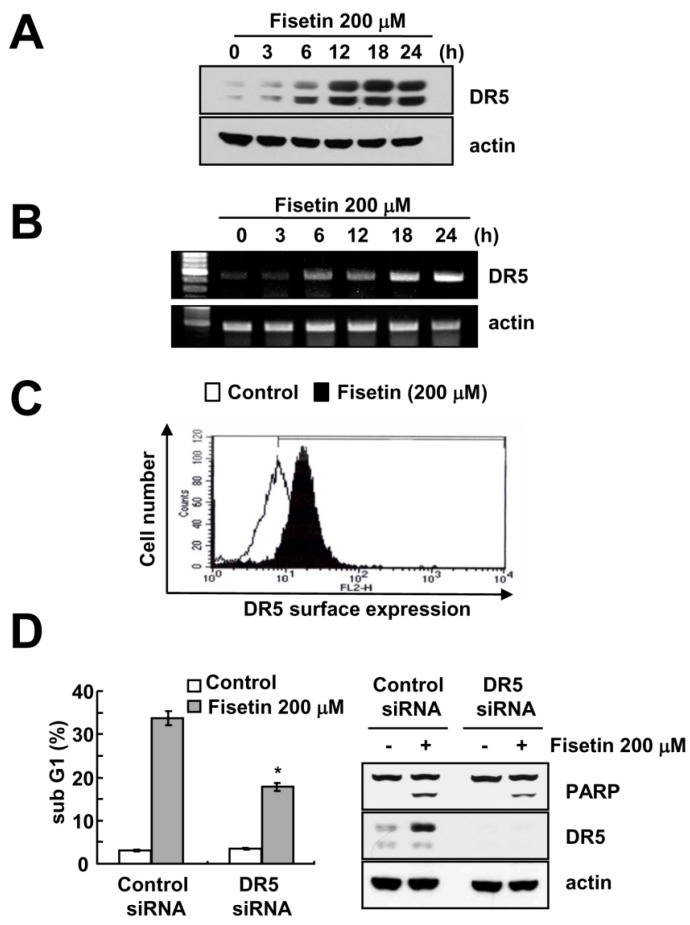
Fisetin induced DR5 expression at a transcriptional level. (**A**,**B**) Caki cells were treated with 200 μM fisetin for the indicated time periods. Western blotting and protein expression determined DR5 mRNA and protein expression, respectively. The level of actin was used as the loading control; (**C**) Caki cells were treated with 200 μM fisetin for 24 h. The cell surface expression level of DR5 was measured by flow cytometry; (**D**) Caki cells were transfected with control or DR5 siRNA. Twenty-four hours after transfection, cells were treated with 200 μM fisetin for 24 h. The level of apoptosis was analyzed by the sub-G1 fraction using flow cytometry. The protein expression levels of PARP, DR5 and actin were determined by western blotting. The level of actin was used as a loading control; the values in (**C**) represent the mean ± SD from three independent samples. * *p* < 0.01 compared to fisetin-treated control siRNA.

Since up-regulation of DR5 expression is induced at significant levels with fisetin treatment, we focused on the modulation of DR5 expression. To confirm the up-regulation of DR5 by fisetin, we examined the effect of fisetin on DR5 expression through the use of a time-kinetic analysis. As shown in Figure 3A, fisetin induced up-regulation of DR5 within 6 h, with regulation gradually increasing up to 24 h.

Furthermore, fisetin modulated DR5 expression on a transcriptional level (Figure 3B). Since translocation of the DR5 protein to the plasma membrane is important for DR-mediated apoptosis, we examined whether fisetin increases DR5 expression at the cell surface. The expression levels of DR5 were higher in fisetin-treated cells compared with that of control cells (Figure 3C). To identify the functional role of DR5 up-regulation of fisetin-induced apoptosis, Caki cells were transfected with DR5 siRNA. Down-regulation of DR5 by siRNA reduced sub-G1 population and cleavage of PARP in fisetin-treated cells (Figure 3D). Therefore, up-regulation of DR5 plays critical roles on fisetin-induced apoptosis.

### 2.4. Endoplasmic Reticulum Stress Has No Effect on Fisetin-Induced DR5 Expression

**Figure 4:**
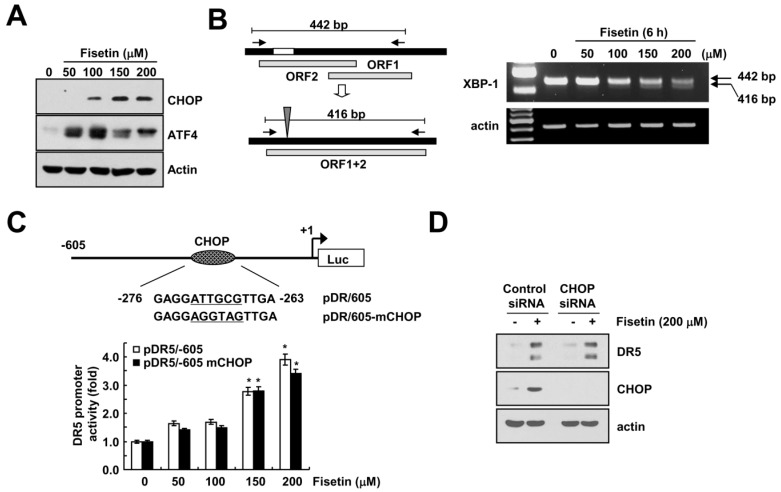
Up-regulation of CHOP expression has no effect on DR5 expression in fisetin-treated cells. (**A**,**B**) Caki cells were treated with the indicated concentrations of fisetin for 6 h; (**A**) The protein expression levels of CHOP, ATF4 and actin were determined by western blotting; (**B**) The mRNA expression levels of XBP-1 and actin were determined by RT-PCR. The level of actin was used as a loading control; (**C**) Caki cells were transiently transfected with pDR5/-605 or CHOP-mutated pDR5/-605 and then treated with the indicated concentrations of fisetin for 24 h. After treatment, cells were lysed and assayed for luciferase activity; (**D**) Caki cells were transfected with control or CHOP siRNA. Twenty-four hours after transfection, cells were treated with 200 μM fisetin for 24 h. The protein expression levels of DR5, CHOP and actin were determined by western blotting. The level of actin was used as a loading control; the values in (**C**) represent the mean ± SD from three independent samples. * *p* < 0.01 compared to control.

Transcriptional regulation of DR5 is mediated by several transcriptional factors. Among them, CCAAT-enhancer-binding protein homologous protein (CHOP) is one of candidates, which modulate DR5 expression [14]. Kang et al. have reported that fisetin induced apoptosis in human non-small cell lung cancer via induction of endoplasmic reticulum (ER) stress [7]. Therefore, we investigated whether fisetin induced ER stress response in human renal carcinoma Caki cells. Fisetin induced expression of ER stress-related proteins, including CHOP and activating transcription factor (ATF4) (Figure 4A). In addition, fisetin also increased the spliced form of the X-box binding protein (XBP)-1 mRNA (Figure 4B).

Next, we examined whether fisetin-induced CHOP expression is involved in DR5 expression. As shown in Figure 4B, we mutated the potential CHOP site (−276 to −263) of the DR5 promoter gene followed by the transfection of Caki cells with either the DR5 promoter (pDR5/605) or mutated CHOP binding site (pDR5/605-mCHOP). The resulting promoter activities were analyzed by a luciferase assay in the presence or absence of fisetin. Fisetin markedly increased the two promoter activities for DR5 (Figure 4C). Furthermore, down-regulation of CHOP by siRNA did not change fisetin-induced DR5 expression (Figure 4D). Therefore, fisetin induced DR5 up-regulation in a CHOP-independent manner.

### 2.5. Fisetin-Induced p53 Expression Is Associated with DR5 Up-Regulation

**Figure 5:**
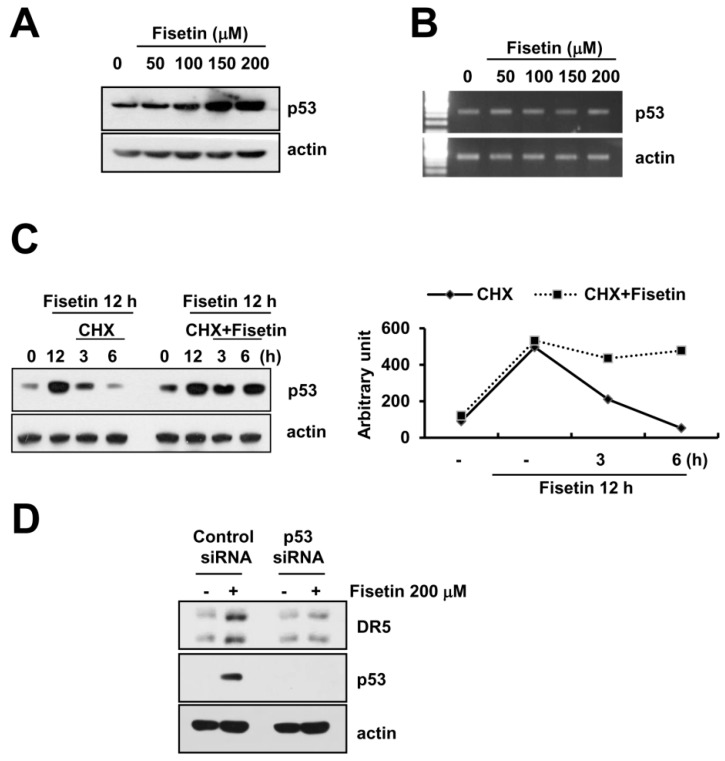
Fisetin induced DR5 expression via up-regulation of p53 expression at the post-translational levels. (**A**,**B**) Caki cells were treated with the indicated concentrations of fisetin for 24 h. p53 and actin protein and mRNA expression were determined by western blotting and RT-PCR, respectively; (**C**) Caki cells were treated with 200 μM fisetin for 12 h, and then washed twice with PBS. After washing, cells were treated with or without 200 μM fisetin in the presence of cyclohexamide (CHX) (20 μg/mL) for the indicated time periods. p53 and actin protein expression were determined by western blotting; the band intensities of p53 protein were measured using the public domain JAVA image-processing program ImageJ (**D**) Caki cells were transfected with control or p53 siRNA. Twenty-four hours after transfection, cells were treated with 200 μM fisetin for 24 h. The protein expression levels of p53, DR5, and actin were determined by western blotting. The level of actin was used as a loading control.

A previous study reported that fisetin induced p53 expression in human colon cancer cells [22]. Additionally, p53 is known to have an important role in the up-regulation of DR5 [23]. Fisetin markedly induced p53 protein expression in a dose-dependent manner (Figure 5A). However, levels of p53 mRNA did not alter in fisetin-treated cells (Figure 5B). Therefore, we investigated whether fisetin modulates p53 protein stability.

Caki cells were treated with cycloheximide (CHX), an inhibitor of de novo protein synthesis in the presence or absence of fisetin. CHX alone gradually decreased p53 protein expression, whereas co-treatment with CHX and fisetin sustained p53 protein expression (Figure 5C). This data indicated that fisetin increased p53 protein expression via induction of protein stability. Next, to identify the significance of fisetin-induced p53 expression, Caki cells were transfected with either the control or the p53 siRNA. Down-regulation of p53 by siRNA markedly inhibited fisetin-induced DR5 expression. Therefore, our data indicated that fisetin induced DR5 expression though up-regulation of p53 protein expression.

### 2.6. Fisetin-Induced Reactive Oxygen Species Production Has No Effect on Apoptosis

**Figure 6:**
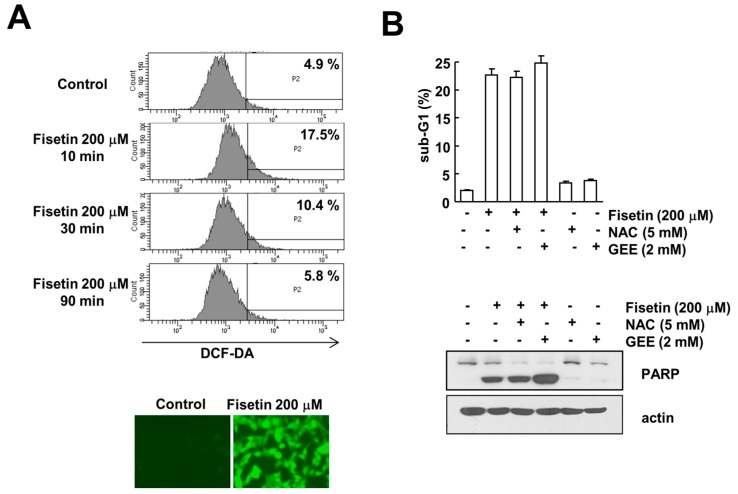
The effect of ROS production on fisetin-induced apoptosis. (**A**) Caki cells were treated with 200 μM fisetin for the indicated time periods (upper panel) or 10 min (lower panel), and were loaded with H_2_DCF-DA fluorescent dye. H_2_DCF-DA fluorescence intensity was detected by flow cytometry (upper panel) and fluorescence microscopy (lower panel); (**B**) Caki cells were pretreated with 5 mM *N*-acetylcystine (NAC) and 2 mM glutathione ethyl ester (GEE) for 30 min, and then stimulated with 200 μM fisetin for 24 h. The sub-G1 fraction was measured by flow cytometry as an indicator of the level of apoptosis. The protein expression levels of PARP and actin were determined by western blotting; the values in (**B**) represent the mean ± SD from three independent samples.

Reactive oxygen species (ROS) is one of the major signaling molecules known to induce apoptosis in cancer cells. Fisetin has been shown to induce ROS production in multiple myeloma, resulting in apoptosis [24]. Therefore, we examined the effect of ROS production on fisetin-induced apoptosis. Fisetin induced ROS production within 10 min, and then gradually decreased to basal levels (Figure 6A). However, ROS scavengers [*N*-acetylcystine (NAC) and glutathione ethyl ester (GEE)] had no effect on fisetin-induced induction of sub-G1 population and PARP cleavage (Figure 6B). Therefore, this data indicated that ROS signaling is not involved in fisetin-induced apoptosis. Taken together, our results demonstrate that fisetin induces apoptosis through p53-mediated up-regulation of DR5 expression in human renal carcinoma Caki cells.

## 3. Discussion

In this study, we demonstrated a mechanism underlying fisetin-induced apoptosis. Fisetin markedly induced apoptosis via induction of DR5 expression. In addition, fisetin induced up-regulation of p53 expression at the post-translational level, and down-regulation of p53 by siRNA-inhibited up-regulation of DR5 expression in fisetin-treated cells. Therefore, our results suggest that fisetin induces apoptosis though p53-mediated up-regulation of DR5 expression.

Several papers have reported that fisetin induced apoptosis in multiple cancer cell lines, and in addition suggested the molecular mechanisms related with induction of apoptosis [7,22,24,25,26,27,28,29]. First, fisetin modulates the MAPK signaling pathway. Fisetin induced apoptosis in human non-small lung cancer cells and cervical cancer cells through activation of the MAPK signaling pathway [7,25]. Sustained activation of ERK and ERK-mediated CHOP expression is associated with fisetin-induced apoptosis [7,25]. Second, fisetin inhibited NF-κB signaling pathways. Fisetin induced apoptosis in colon cancer cells as well as in bladder cancer cells through inhibition of NF-κB signaling pathways [26,27]. Inhibition of NF-κB signaling resulted in down-regulation of anti-apoptotic proteins (c-FLIP and cyclooxygenase-2). Third, p53 plays critical roles on fisetin-induced apoptosis. Two previous studies reported that induction of p53 expression contributed to induction of apoptosis in human colon cancer cells [22] and bladder cancer cells [27]. In contrast, fisetin can also induce apoptosis in a p53-independent manner. In gastric cancer cells, although fisetin increased p53 protein expression, inhibitor of p53 did not reduce fisetin-mediated apoptosis [3]. Therefore, roles of p53 on fisetin-induced apoptosis are different depending on cell type. Finally, ROS are important signaling molecules on fisetin-induced apoptosis. Production of ROS by fisetin treatment is associated with induction of apoptosis in multiple myeloma [24] and oral cancer cells [8]. An ROS scavenger molecule, NAC, reduced fisetin-induced apoptosis in both cells. Recently, Wu et al. suggested that NAC enhanced fisetin-induced apoptosis through inhibition of ERK phosphorylation in colon cancer cells [28]. In this study, NAC completely inhibited H_2_O_2_-induced ROS production and apoptosis, whereas fisetin-induced ROS production did not alter with NAC treatment [28]. Lim et al. suggested that the enhanced effect of NAC on fisetin-mediated apoptosis is independent of the scavenging effect of NAC. In our study, NAC did not enhance fisetin-induced apoptosis, and the ROS scavenger, GEE, also had no effect on apoptosis (Figure 6B). Therefore, ROS signaling is not associated with fisetin-induced apoptosis in human renal carcinoma Caki cells.

As shown in Figure 2C and Figure 3C, fisetin induced DR5 expression and subsequently increased DR5 presentation on cell surface. Previous studies reported that death receptors could activate in a ligand-independent manner [29,30,31]. For example, palmitate increased ER stress-induced CHOP expression, resulting in the upreguatlion of DR5 in hepatocytes [29]. Although human recombinant DR5-Fc chimera proteins inhibited TRAIL-induced apoptosis, plamitate-induced apoptosis through induction of DR5 was not blocked by DR5-Fc [29]. In addtion, persistent ER stress-mediated up-regulation of DR5 expression also promoted apoptosis through ligand-independent DR5 activation [30]. Furthermore, Lim et al. reported that the recruitment of DR5 to a lipid raft induced DR5 activation, followed by apoptosis in ursodeoxycholic acid-treated human gastric cancer cells [31]. Therefore, this strategy to induce up-regulation of DR5 might be effective to promote cancer cell death. Taken together, our data suggested that fisetin induced apoptosis through p53-mediated up-regulation of DR5 expression in human renal carcinoma Caki cells, and these findings provide an important molecular mechanism involved in fisetin-induced apoptosis.

## 4. Materials and Methods

### 4.1. Cell Culture and Materials

Human renal carcinoma (Caki), human hepatoma (SK-Hep1), and mouse kidney (TCMK-1) were obtained from the American Type Culture Collection (Manassas, VA, USA). The culture medium used throughout these experiments was Dulbecco’s Modified Eagle’s Medium (DMEM) containing 10% fetal bovine serum (FBS), 20 mM HEPES buffer and 100 μg/mL gentamycin. The PCR primers were purchased from Macrogen (Seoul, Korea). The z-VAD-fmk was purchased from R&D Systems (Minneapolis, MN, USA), and *N*-acetyl-L-cysteine (NAC) was obtained from Calbiochem (San Diego, CA, USA). Anti-Bcl-2, anti-p53, anti-DR4, anti-Bcl-xL, anti-PUMA, anti-CHOP, and anti-ATF4 antibodies were purchased from Santa Cruz Biotechnology (Dallas, TX, USA). Anti-DR5 and anti-PARP antibodies were obtained from Cell Signaling Technology (Beverly, MA, USA). Anti-c-FLIP antibody was obtained from ALEXIS Corporation (San Diego, CA, USA). Anti-FAS antibody was obtained from Upstate-Merck Millipore (Lake Placid, NY, USA). Anti-actin antibody and other chemicals were obtained from Sigma Chemical Co. (St. Louis, MO, USA).

### 4.2. Flow Cytometry Analysis

For flow cytometry, the cells were resuspended in 100 μL of phosphate-buffered saline (PBS), and 200 μL of 95% ethanol was added while the cells were being vortexed. Next, the cells were incubated at 4 °C for 1 h, washed with PBS, resuspended in 250 μL of 1.12% sodium citrate buffer (pH 8.4) with 12.5 μg of RNase and incubated for an additional 30 min at 37 °C. The cellular DNA was then stained by adding 250 μL of a propidium iodide solution (50 μg/mL) to the cells for 30 min at room temperature. The stained cells were analyzed by fluorescent-activated cell sorting on a FACSCanto II flow cytometer (BD Biosciences, San Jose, CA, USA) to determine the relative DNA content, which was based on the red fluorescence intensity.

### 4.3. Western Blot Analysis

Cells were washed with cold PBS and lysed on ice in modified RIPA buffer (50 mM Tris-HCl, pH 7.4, 1% NP-40, 0.25% Na-deoxycholate, 150 mM NaCl, 1 mM Na_3_VO_4_, and 1 mM NaF) containing protease inhibitors (100 μM phenylmethylsulfonyl fluoride, 10 μg/mL leupeptin, 10 μg/mL pepstatin, and 2 mM EDTA). Lysates were centrifuged at 13,000× *g* for 15 min at 4 °C, and the supernatant fractions were collected. Proteins were separated by SDS-PAGE and transferred to an Immobilon-P membrane. Specific proteins were detected using enhanced chemiluminescence.

### 4.4. 4′,6′-diamidino-2-phenylindole Staining (DAPI) for Nuclei Condensation and Fragmentation

To examine cellular nuclei, the cells were fixed with 1% paraformaldehyde on glass slides for 30 min at room temperature. After fixation, the cells were washed with PBS and a 300 nM 4′,6′-diamidino-2-phenylindole solution (Roche, Mannheim, Germany) was added to the fixed cells for 5 min. After the nuclei were stained, the cells were examined by fluorescence microscopy.

### 4.5. DNA Fragmentation Assay

The cell death detection ELISA plus kit (Boerhringer Mannheim; Indianapolis, IN, USA) was used to determine the level of apoptosis by detecting fragmented DNA within the nuclei of fisetin-treated cells. Each culture plate was centrifuged for 10 min at 200× *g*, the supernatant was removed, and the cell pellet was lysed for 30 min. Next, the plate was centrifuged again at 200× *g* for 10 min, and the supernatant that contained the cytoplasmic histone-associated DNA fragments was collected and incubated with an immobilized anti-histone antibody. The reaction products were incubated with a peroxidase substrate for 5 min and measured by spectrophotometry at 405 nm and 490 nm (reference wavelength) with a microplate reader. The signals in the wells containing the substrate alone were considered as background and were subtracted.

### 4.6. Asp-Glu-Val-Asp-ase (DEVDase) Activity Assay

To evaluate DEVDase activity, cell lysates were prepared after their respective treatments with fisetin. Assays were performed in 96-well microtiter plates by incubating 20 μg of cell lysates in 100 μL of reaction buffer (1% NP-40, 20 mM Tris-HCl, pH 7.5, 137 mM NaCl, 10% glycerol) containing a caspase substrate [Asp-Glu-Val-Asp-chromophore-*p*-nitroanilide (DVAD-pNA)] at 5 μM. Lysates were incubated at 37 °C for 2 h. Thereafter, the absorbance was measured at 405 nm with a spectrophotometer.

### 4.7. Analysis of Cell Surface DR5

Cells were detached with 0.5 mM EDTA, and washed three times with PBS wash buffer supplemented with 0.5% BSA. Cells (5 × 10^5^). Cells were resuspended in 200 μL of PBS, and stained with primary antibody (1 μg/mL) and incubated for 30 min at 4 °C. Unreacted antibody was removed by washing the cells twice with the same PBS buffer. Cells were stained with the secondary antibody conjugated with fluorescein isothiocyanate (FITC) and incubated for 30 min at 4 °C. Unbound FITC-conjugated antibody was washed twice with PBS. Cells were resuspended in 200 μL of PBS. Cell-surface expression of DR5 receptor was determined by flow cytometry. Fluorescent intensity of the cells is directly proportional to the density of the present receptor.

### 4.8. Reverse Transcription Polymerase Chain Reaction (RT-PCR)

Total RNA was isolated using TriZol (Life Technologies; Gaithersburg, MD, USA), and the cDNA was prepared using M-MLV reverse transcriptase (Gibco-BRL; Gaithersburg, MD, USA) according to the manufacturers’ instructions [32,33]. The following primers were used for the amplification of human p53, DR5, Xbp-1, and actin: p53 (sense) 5′-GAA GAC CCA GGT CCA GAT GA-3′ and (antisense) 5′-CTC CGT CAT GTG CTG TGA CT-3′; DR5 (sense) 5′-AAG ACC CTT GTG CTC GTT GT-3′ and (antisense) 5′-GA CAC ATT CGA TGT CAC TCC A-3′; Xbp-1 (sense) 5′-CCT TGT AGT TGA GAA CCA GG-3′ and (antisense) 5′-GGG GCT TGG TAT ATA TGT GG-3′; actin (sense) 5′-GGC ATC GTC ACC AAC TGG GAC-3′ and (anti-sense) 5′-CGA TTT CCC GCT CGG CCG TGG-3′. The PCR amplification was carried out using the following cycling conditions: 94 °C for 3 minutes followed by 17 cycles (actin) and 25 cycles (p53 and Xbp-1) or 28 cycles (DR5) of 94 °C for 45 s, 58 °C for 45 s, 72 °C for 1 min, and a final extension at 72 °C for 10 min. The amplified products were separated by electrophoresis on a 1.5% agarose gel and detected under UV light.

### 4.9. DNA Transfection and Luciferase Assay

Transient transfection was performed on six-well plates. One day before transfection, Caki cells were plated to maintain approximately 60–80% confluence. DR5 promoter and mutant CHOP containing DR5 promoter plasmid were transfected into cells using the Lipofectamine™ 2000 reagent (Invitrogen, Carlsbad, CA, USA). To assess promoter-driven expression of the luciferase gene, cells were collected and disrupted by sonication in lysis buffer (25 mM Tris-phosphate, pH 7.8, 2 mM EDTA, 1% Triton X-100, and 10% glycerol), and aliquots of the supernatants were analyzed by measuring luciferase activity according to the manufacturer’s instructions (Promega, Madison, WI, USA).

### 4.10. Small Interfering RNA (siRNA)

The DR5, p53 and CHOP siRNA used in this study were purchased from Santa Cruz Biotechnology. The green fluorescent protein (GFP) (control) siRNA was purchased from Bioneer (Daejeon, Korea). Cells were transfected with siRNA using Oligofectamine Reagent (Invitrogen) according to the manufacturer’s recommendations.

### 4.11. Measurement of Reactive Oxygen Species (ROS)

Intracellular accumulation of ROS was determined using the fluorescent probes 2′,7′-dichlorodihydrofluorescein diacetate (H_2_DCFDA) and Mitosox Red. Caki cells were treated with fisetin, and then cells were stained with the H_2_DCFDA fluorescent dye or Mitosox Red for an additional 10 min. Cells were then trypsinized and resuspended in PBS, and fluorescence was measured at specific time intervals with a flow cytometer (Becton–Dickinson; Franklin Lakes, NJ, USA) or fluorescence microscopy (Zeiss, Jena, Germany).

### 4.12. Statistical Analysis

The data was analyzed using a one-way ANOVA and post hoc comparisons (Student-Newman-Keuls) using the Statistical Package for Social Sciences 22.0 software (SPSS Inc.; Chicago, IL, USA). We determined the sample size on the basis of the smallest effect we wish to measure.

## Abbreviations

ROS	reactive oxygen species
TRAIL	tumor necrosis factor-related apoptosis-inducing ligand
DR	death receptors
CHOP	C/EBP homologous protein
ER	endoplasmic reticulum
CHX	cycloheximide
